# External Urethrotomy with Cases*Read before the Baltimore Medical and Surgical Society.

**Published:** 1882-01

**Authors:** Charles F. Bevan

**Affiliations:** Professor of Anatomy and Genito-Urinary Surgery, College of Physicians and Surgeons, Baltimore; Md.


*FOR CONTENTS OF THIS NUMBER SEE PAGE 72.*

*THE*

### Independent Practitioner.

VOL. III.——*January, 1882.*——NO. 1.

### ORIGINAL COMMUNICATIONS.

*We are not responsible for the opinions expressed by contributors.*

ARTICLE I.

### EXTERNAL URETHROTOMY, WITH CASES.*

BY CHARLES F. BEVAN, M. D ,
Professor of Anatomy and Genito-Urinary Surgery, College of Physicians and Surgeons, Baltimore, Md.

Any one who has gone through with the trials and annoyances attendant upon an ugly case of retention of urine; who has escaped from that perplexing question, "what else shall I do," will be apt to sympathize most cordially with those who have had similar experiences. In the hope of gathering the methods and results which have been practiced by the various members of this society, I have thought it might be profitable to relate such cases of my own as have demanded surgical aid, especially the severe procedure of perineal section, both with and without the guide.

Accumulations of urine are generally due to the presence of stricture of the urethra at some part or parts of its course, and are either spasmodic or organic in character. Discarding those cases in which the element of spasm is the chief factor, and such of the organic class as are amenable to the medical art, there will remain only a small number, I imagine. which will fall to the lot of each in-

*Read before the Baltimore Medical and Surgical Society.

dividual practitioner. It will be from the "minority" however that the troubles arise. It will be found that the stricture in these cases is generally of long standing, with, perhaps, very serious alterations in the urethral canal often due to inflammatory and cicatricial alterations, and often complicated with false passages; for the victims playing doctor for themselves have had fools for their patients, and their urethræ are monuments to their bad surgery. Occasionally one encounters a case due to traumatism; from blows, falls or other injuries to the perineum and adjacent parts, a rupture of the urethra usually transverse is met with, and these cases are always grave. The attack of retention, is preceded by a slow and gradual diminution of the stream requiring greater expulsive efforts on the part of the bladder, leading to hypertrophy of its muscles. Occasionally the retention is only temporary and a few hours of rest suffice to start the stream; later on in the progressive history of the stricture, however, the canal becomes more and more narrow and finally some dietetic or barometric change adds the last straw, the camel's back is broken and the water will not pass. Distention of the bladder from accumulation then takes place. Relief is obtained either by a very gradual oozing occurring, the urine passing guttatim, or else by dilatation of the urethra behind the contraction until at length rupture of its walls takes place, with extravasation. Prior to extravasation, the surgeon who has pinned his faith to Syme's dictum, "that whenever urine passes outwards through a stricture, an instrument ought with care and perseverance to be got in," will be often sorely perplexed as to what to do. The degree of skill possessed by medical men in the passage of urethral instruments is various; and while I realize that the belief in Syme's dictum has been often, in extreme cases, verified greatly to the benefit of patient and practitioner, I am also equally well satisfied that one's perseverance is not always rewarded and that valuable time is often lost, to the patient's detriment. The following cases are, I think, to the point:

Case I. P. T. U.,—age 39—was first seen April 7th, 1879, in consultation with Dr. Z. K. Wylie. The history of the case is briefly as follows:—Some 15 years ago had gonorrhœa for the first time; has had repeated attacks since; has noticed a gradual diminution in the size of his stream, difficulty in voiding it, and several attacks of retention. Was operated upon by Dr. Claudius H. Mastin, of Mobile, in 1872, who he thinks divided the constriction upon the Maisonneuve instrument; for several years after this he got along very well, his only annoyance being an obstinate gleet; of late his old trouble has returned so that he now voids his urine drop by drop, and his bladder is generally full. Penis measures 3½ inches in circumference, estimated urethra 34 mm. Urethrametre was arrested at 6½ inches, from meatus; there was a uniform calibre to urethra in front of stricture equal to 35 mm., except at the meatus which was contracted to 28 mm.

For *one month* the patient was confined to bed, treated with hot baths and fomentations daily, whilst both with and without chloroform, repeated, persistent and prolonged efforts with every imaginable urethral instrument were resorted to to pass the stricture, but to no purpose. May 12th, assisted by Drs. Wylie, Coskery, Latimer and others, I proceeded to perform the perineal section without a guide. An incision of 2½ to 3 inches was made in the median line of the perineum and the urethra opened behind the stricture; a sound was then carried down to the front face of the stricture, was depressed, and after searching for the opening with the grooved director in vain, the stricture was divided till the sound was reached. The full-sized sound, 35 F., was carried to the bladder, and after its withdrawal, was replaced by a catheter which was tied in. Time of operation, 1 hour and 20 minutes.

Twelve hours after the operation the patient had a severe chill followed by nausea, vomiting and high fever; the thermometer ranging from 102°–104° F., with a pulse of 120–160. This condition lasted some two or three days, and is believed to have been largely due to the presence in the bladder of the catheter, for upon its withdrawal the nausea, vomiting and fever subsided. May 16th, the perineal wound seemed to be doing well, but the scrotum commenced to swell and in a day or two cellulitis made its appearance involving the wound and posterior parts of the scrotum, and a small slough occurred. May 24th—part of the urine passed through penis; four days later all of it was voided per urethram. The sound, 35 F., was passed regularly every 4 days up to the latter part of June One year after operation, and again in October, 1881, on examination with the full-sized sound no obstruction can be made out.

Case II. E. C.,—age 57—by occupation a cutler—came under observation at the City Hospital, August 10th, 1879, suffering from retention of urine. He gave at once a history of urethral stricture of long standing produced by gonorrhœa. Twenty years ago had undergone a . operation at the University College Hospital, London, performed y Sir Hy Thompson. For years has been in the habit of passing a catheter 2 or 3 times a month, and had found it necessary to diminish the size of the instrument. Passed a No. 2 F. some 6 weeks prior to entering the hospital, but had not been able since then to introduce anything.

Upon examination his penis measured 3¼ inches in circumference; estimated size of urethra 32 F. A condition of congenital hypospadias existed, the urethral orifice opening at the corona glandis and being of small size (27 F). A sound was passed to bulbo-membranous junction; several false passages exist anterior to stricture, the existence and cause of which were known by the patient who possessed great cleverness in catheter manipulation. Examination by rectum showed prostate very much enlarged. Systematic efforts were made to effect an entrance to bladder in vain; the condition of retention, however, was overcome by hot baths, rest and leeches to perineum. Patient left the hospital August 20, attending, however, twice each week as an out-door patient; efforts to introduce instruments were thus kept up until November 6th, at which time he returned to the hospital, and on November 8th, assisted by Prof. Oscar J. Coskery and others, the operation of perineal section without a guide was performed. After exposing the urethra posterior to the stricture, the sound was introduced through the penis to the anterior surface of the contraction, was depressed and the cicatricial material divided, allowing the sound to pass out at the lower opening. It was impossible to carry it, however, into the bladder owing to the size of the prostate and the faulty curve of the sound, and as it was seen that urine escaped through the wound, he was sent back to bed. Time of operation 45 minutes. November 9th, had severe chill, followed by fever—temperature reaching 103°–105° F., falling in a few hours. November 12th, doing well in all respects, full-sized sound passed without difficulty into bladder. November 28th, passed his water through penis; two days later he returned to his home continuing his visits as an out-patient. November 30, patient well, wound closed, sound 32 F., passes readily to bladder. Reports from time to time at 2 months' intervals for inspection. November 10, 1881, passed 32 F.

In each of these two cases it will be seen, that while the urethra was pervious to urine it resisted the passage down it of any instrument. In the first case over one month was consumed in perseverance, and about 3 months in the second. The greatest care was exercised in both, and the talents of quite a number of medical gentlemen were taxed in fruitless efforts to restore the natural canal without resorting to the perineal section without a guide. Whenever the urine can escape even in very minute quantities, although distention of the bladder may take place giving rise to no inconsiderable amount of pain and inconvenience, it rarely happens that the integrity of the urethra, perineum or bladder is seriously jeopardized. A sufficient amount of time may be spent in attempting to reach the bladder, and so either dilate the obstruction with instruments or permit the perineal section to be done upon a guide. When, however, dilatations of the urethra behind the stricture occur, when these parts become thin and softened either by inflammation or the dilating process, or where the urethra has been ruptured, the condition of retention is apt to be promptly followed by extravasation, with the formation of urinary fistulæ. These openings are troublesome in the extreme, unfitting the individual for any social relations or business and rendering life a burden.

Case III. Isaac B., colored—age 47—laborer—consulted me first about 25th February, 1878, at the City Hospital Dispensary, complaining of an inability to pass his water; had experienced more or less difficulty for 2 or 3 years, during which time the stream had become very small. Had had several attacks of retention before, which had been relieved by rest, hot baths and opium; the present attack of 12 hours duration had not yielded to the old treatment. His first case of gonorrhœa occurred 18 or 20 years ago, and he had had a gleet ever since.

On examination his penis was found to have a circumference of $3\frac{3}{4}$ inches; estimated urethra, 36 inches. The urethra-metre was arrested at 3 inches from the meatus, anterior to obstruction; the calibre of the urethra was 36 inches, narrowing to 18 inches at the meatus. A small sound, No. 4 F., passed the obstruction at 3 inches, but was arrested at the bulbo-membranous junction; trials with fine flexible and filiform instruments were made without result. The patient was then ordered to enter the hospital, which he promised and failed to do. Three days later, February 28, he applied for admission, and when examined it was found that extravasation had taken place. The penis was distended, hard and inflamed ; scrotum about the size of a fœtal head, hard and very tense. General condition very bad, due to a rapidly developing cellulitis. Incisions were at once made in the perineum, one in the median line and one on each side of it, giving exit at once to considerable oozing of fluid, ammoniacal in odor, &c. In the course of three weeks it was found that whilst a large slough had occurred, the parts had begun to granulate and cicatrize, and that through the central opening *all* of his urine was voided. Efforts were now made to restore the urethral canal ; the sound could not be carried beyond the bulbo-membranous junction. Under chloroform the perineal opening was enlarged, and by a director the sound previously carried down the urethra was reached. The anterior contraction and meatus were divided by the dilating urethrotome and bistoury, and the 36 F. sound introduced to the bladder. From this time on his recovery was rapid, the sound was regularly passed, perineal wound was closed, and he was discharged voiding his water naturally, about the last of April. 18 months later the 36 F. sound was passed without meeting an obstruction.

Case IV. J. S.,—age 43—married—consulted me for urinary fistula on July 21st, 1879. He gave the history of repeated attacks of gonorrhœa 20 years ago, and had several attacks of retention, one of which was followed by extravasation, abscesses and fistulæ. He had had the perineal section performed successfully, and for some time afterwards his condition had been excellent. His old trouble had, however, returned, and when examined by me, three openings existed in the perineum, through which all of his water passed. The penis measured 3½ inches in circumference, with an estimated urethra of 34 mm. A No. 2 F. sound detected a dense obstruction at the bulbo-membranous junction, but the bladder could not be reached with any larger instrument. His urine contained pus, mucus, albumen and granular tube casts, while the rational symptoms of Bright's disease were well marked. Under chloroform the urethra was dilated, the dilating urethrotome introduced, and the stricture was freely divided : the fistulæ were then divided so as to communicate freely with the urethra. The 34 F. sound was passed every 3 or 4 days. In the course of 3 or 4 weeks, the perineal wounds which had been dressed with carbolized oil and lint, gradually healed, allowing the urine to escape through the natural channel. His vesical trouble which had before the operation, been very annoying, now subsided, leaving him in a far more comfortable and decidedly improved condition. His urine, however, continued to show the evidences of the kidney lesion, which finally produced death twenty months after the last operation. A post-mortem could not be obtained, much to my regret: within a week, however, prior to his death, the 34 F. sound passed easily to the bladder.

Case V. E. C. W.,—age 65—came under observation September 1st, 1881, in consultation with Prof. A. F. Erich. The history as given is, that up to January, 1881, he had never experienced any urethral or vesical disorder; had had remarkably fine health, &c.; in January had a fall, the limbs separating widely; at once experienced a feeling as if something had given way; sharp pain in perineum, &c. Some hours afterwards found himself unable to pass his urine. A few drops and considerable blood escaped later on, when after a hot hip bath the effort was repeated. For two or three days this difficulty continued, only a few drops at a time escaping, when he discovered a sensitive lump in the perineum. The swelling continuing to increase, as also did the pain, he consulted Prof. Erich. The perineal mass was opened, giving exit to pus and urine: several other abscesses formed from time to time, healing in one place and opening in another, but always producing fistulæ through which his water passed, only a small part traversing the urethra. When examined, September 1st, his perineum contained numerous,—5 to 7, fistulous openings communicating with each other, or with the urethra. Circumference of penis, $3\frac{1}{4}$ inches; estimated urethra 36 mm. Urethrametre passed to bulbo-membranous region, and when expanded located a contraction at the meatus and first half inch to 23 mm. The contraction at $5\frac{1}{2}$ inches admitted a No. 12 F. Under ether and with the assistance of Drs. Erich and Rohé, I succeeded in passing the dilating urethrotome, and with it divided the contraction, allowing the sound to pass to the bladder. Three days later with the sound again in the bladder, a probe was carried through the fistulæ: those communicating with each other were so cut as to make one tract; such as communicated with the urethra were then divided so as to make two large openings in the perineum, communicating with the urethra. The wounds were then packed with lint, previously soaked in carbolized oil. The case progressed from this time in the most favorable manner. September 10th, urine passes in about equal quantity through penis and perineum. September 20, five-sixths through penis, and by the first of October the deeper parts of the fistula had closed, all urine going through the natural channel. Cicatrization gradually closed the wounds, and now his condition is excellent. The 36 sound passes unobstructed to the bladder. His general health, wretched at the time of operation, is now greatly improved and he may be classed as thoroughly well.

*Indications for the Operation.*—The vast majority of all strictures met with are amenable to less heroic measures than the perineal section; gradual and continuous dilatation; or dilating urethrotomy suffice for the majority of all cases whether located in the anterior or posterior part of the urethra. It should be accepted as a rule, that perineal section should not be resorted to whenever any instrument can be introduced into the bladder. But when the stricture is impassable, even though permeable to the urine, especially if of traumatic origin, no method of treatment will yield as gratifying results, and of as permanent a character as the perineal section. The operation is, however, a formidable one, requiring abundance of good light, slow work and an intimate acquaintance with the anatomy of the parts.

Where fistulæ are present, where the urethra is complicated with false passages, the operation introduced by Syme, viz.: Cutting upon a guide should be practiced. The essential here is, that the bladder should be reached, and this may be done by instruments passed through the urethra, or by utilizing the fistulous openings.

In utilizing the fistulæ the sound should be passed to the stricture and through it when possible; long grooved directors may then be passed through the openings until the sound is reached and the section made upon the director. In either case after completion of the work, no instrument should be tied in the bladder: the doing so serves no good purpose, and does harm in irritating the bladder.

